# Increased Levels of Circulating Iron-Albumin Complexes in Peripheral Arterial Disease Patients

**DOI:** 10.3390/antiox12020503

**Published:** 2023-02-16

**Authors:** Elisabetta Schiano, Enrico Cappello, Domenico Cecere, Francesco Pompeo, Ettore Novellino, Mariano Stornaiuolo, Marcello Izzo

**Affiliations:** 1 Department of Pharmacy, University of Naples Federico II, 80131 Naples, Italy; 2 IRCCS Neuromed, 86077 Pozzilli, Italy; 3 Department of Medicine and Surgery, Università Cattolica del Sacro Cuore, 00168 Rome, Italy; 4 MathTechMed-Department of Mathematics for Technology, Medicine and Biosciences Research Center, University of Ferrara, 44121 Ferrara, Italy

**Keywords:** iron, albumin, oxidative stress, transferrin, NTBI, inflammation, peripheral vascular disease, peripheral arterial disease, PAD, PVD, CVD, CVI

## Abstract

Under physiological conditions, extracellular iron circulates in the blood bound to transferrin. As a consequence of several pathologies, the circulating level of a Non-Transferrin Bound pool of Iron (NTBI) increases. The NTBI pool is biologically heterogeneous and represented by iron chelated either by small metabolites (citrate, amino acids, or cofactors) or by serum proteins. By promoting reactive oxygen species (ROS) and reactive nitrogen species (RNS) formation, NTBI causes oxidative stress and alteration of membrane lipids, seriously compromising the healthy state of organs and tissues. While NTBI involvement in several pathologies has been clarified, its contribution to vascular diseases remains to be investigated. Here we measure and analyze the pool of NTBI in the serum of a small group of peripheral arterial disease (PAD) patients. We show that: (i) the NTBI pool shifts from low molecular complexes to high-molecular ones in PAD patients compared to healthy controls; (ii) most of this NTBI is bound to the serum protein Albumin; (iii) this NTBI-Albumin complex can be isolated and quantitated following a simple immunoisolation procedure amenable to automation and suitable for clinical screening purposes.

## 1. Introduction

Iron is the fourth most abundant element of the earth’s crust, and its ionic forms (Fe^3+^ ferric and Fe^2+^ ferrous) play pivotal roles in many different essential processes ranging from oxygen transport to cytochrome activity, cellular respiration, nucleic acids metabolism, and epigenetic regulation of gene expression [1]. In circulating plasma, Fe^3+^ and Fe^2+^ are associated with carrier-proteins, mostly transferrin. Under physiological conditions, Plasma Transferrin Saturation (TSAT) by trivalent Fe^3+^ iron reaches 20–35% and involves dimeric transferrin (fully saturated) and monomeric transferrin (bound only to one Fe^3+^). Inside the cell, iron is instead bound to ferritin, heme, myoglobin, etc. Iron transfer from plasma to cells occurs mainly through dimeric transferrin [2]. Lack of transferrin, excessive absorption of iron from the diet, or its excessive release from cellular reserves may lead to an increase in circulating Non-Transferrin Bound Iron (NTBI). In NTBI, iron may bind to proteins like albumin, or be chelated by molecules like citrate, phosphate, or malate. Under physiological conditions, total NTBI does not exceed 1.0 μM and is commonly undetectable [3]. Infections, inflammations, chronic diseases, neoplasms, malnutrition, and nephrotic syndrome can also influence TSAT by reducing the transferrin rate of synthesis. Indeed, a clear correlation exists between the degree of TSAT and the increase in NTBI [4,5,6,7]. Notwithstanding, NTBI has been shown to increase in patients with normal TSAT also. Interestingly, increased NTBI and normal TSAT values are found in patients affected by peripheral arterial (PAD) or vascular (PVD) diseases, atherosclerosis, and diabetes mellitus [7,8,9,10]. 

As consequences of their propensity to generate reactive oxygen (ROS; Fenton and Haber-Weiss reaction) and/or nitrous species (RNS), NTBI species are highly toxic for the vessels. NTBI is directly absorbed by endotheliocytes [11] and promotes lipid oxidation, damaging plasma membrane and intracellular organelles, ultimately leading to PAD and PVD [3,12]. Fertile females have lower iron stores and thus circulating levels of NTBI, which is thought to confer on them protection from iron overload diseases [13].

Despite its pathophysiological relevance [9,14], the exact contribution of the NTBI pool of iron to vascular disease remains largely unknown. The clarification of the NTBI role in PAD is also mandatory, to confirm its significance as a clinical biomarker and to justify its eligibility among the blood parameters (Malondialdehyde, 4-Hydroxynonenal, F2-isoprostanes, β-2 microglobulin, C-reactive protein, cystatin C, Lipoprotein A) that are currently checked in PAD patients.

NTBI has been involved as well ‘in various brain pathologies such as neurodegenerative Alzheimer’s disease and Parkinson’s disease [15,16]. Iron-mediated oxidative damage also appears to be implicated in the genesis of cerebral vasospasm, and in various forms of cerebral hemorrhage. In particular, a possible relationship between NTBI levels in cerebrospinal fluid and the development of brain lesions in patients with Subarachnoid hemorrhage [17] has been hypothesized due to significantly higher levels of NTBI in patients developing delayed ischemia.

Here, we measure and analyze the pool of NTBI in a small group of PAD patients. We show that, despite these patients’ present values of serum iron, transferrin and ferritin falling into physiological ranges, their serums present increased level of NTBI. Interestingly, most of their NTBI is bound to Human Serum Albumin (HSA). Finally, we present an unprecedented procedure of immunoisolation of these NTBI-HSA complexes, amenable to automation and suitable for clinical screening purposes.

## 2. Materials and Methods

### 2.1. Study Population and Design

Study participants were recruited by the Institute for Treatment and Research IRCCS NEUROMED. A letter of intent regarding volunteers, protocol, and synoptic documents of the study were submitted to the Institutional Review Board of NEUROMED. The study was approved by the committee (protocol code 250619 of 25-06-2019) and conducted in accordance with the declaration of Helsinki of 1964 (as revised in 2000). Patients aged 18–95 years of both sexes were eligible for enrollment. For healthy controls, all willing adults that were non-iron-deficient and not affected by chronic diseases, such as diabetes or hypertension, were included in the study. For PAD patients, the diagnosis of arterial occlusive disease of the lower limbs was made on the basis of history and of physical examination. Duplex ultrasound together with imaging allowed localization and quantification of the arterial lesions and quantification of abnormal flow hemodynamics. Intra-arterial digital subtraction arteriography was used to evaluate the lower extremity arterial tree. All the procedures involving ATK (above the Knee) and BTK (below the Knee) vessels were performed by means of antegrade echo-guided or radioscopical femoral puncture, using an 11-cm-long, 5-F introducer sheath. A total of 14 patients with PAD and 10 healthy patients who met the eligible criteria were enrolled. All the participants received written and oral information concerning the study before they gave their written consent.

### 2.2. Serum Sample Collection

Blood samples were collected from patients in fasting states. From each patient, 5–8 mL of whole blood was drawn into vacutainers and centrifuged at 1000× *g* for 20 min at 25 °C to separate blood cells and serum. After collection, serum samples were aliquoted and stored at −80 °C until further analysis. Serum iron and transferrin (Cobas Integra analyser from Roche, Basel, Switzerland), and serum ferritin (AxSym analyzer from Abbott, Chicago, IL, USA) were immediately determined. A colorimetric (Ab932715, AbCam, Cambridge, UK) and an enzyme-linked immunosorbent assay (Ab288174, AbCam, Cambridge, UK) were used to measure total iron-binding capacity (TIBC) following manufacturer instruction. Briefly, Human Anti-Transferrin antibodies were used to measure the amount of transferrin in the sample. In parallel, iron standard solutions were added to serum samples to saturate the transferrin. The amount of iron remaining free was then measured by colorimetry at a neutral pH using deferoxamine mesylate as iron chelator. Total iron was then measured after acidification by HCl. All measurements were used to calculate total iron binding capacity of the biological samples.

### 2.3. Gel Filtration and Purification of Iron Complexes

Serum samples were rapidly defrosted at R.T. and kept on ice until sample processing. Specifically, to isolate low-molecular iron complexes, 200 μL of serum was loaded on a prepacked PD-10 Desalting Column (Amersham) containing 8.3 mL of Sephadex™ G-25 resin column. Columns were pre-equilibrated with Milli-Q water. Unabsorbed high-molecular-weight proteins and complexes were rapidly eluted using water, while the adsorbed low molecular iron complexes were desorbed using sequential addition of 10–20 column volumes of eluent. *Size Exclusion Chromatography*: to isolate and analyze the pool of NTBI contained in high-molecular iron complexes, 100 μL of serum samples were loaded on either an Enrich SEC 70–75 HR 10/300 or an Enrich SEC 650 10/300 gel filtration column (both from BIORAD, Hercules, CA, USA) pre-equilibrated in 0.9% mM NaCl (pH 7.4). Isocratic elution was followed by measuring absorbance at 230 nm. Elution fractions (1 mL) were analyzed in terms of iron content (calcein measurement), protein content (Bradford assay), and resolved by SDS-PAGE followed by Coomassie staining [18]. Standardization of the analytical method was achieved by using pure HSA (lyophilized powder, fatty acid free, globulin free, ≥99%) (agarose gel electrophoresis, A3782, Sigma-Aldrich (Milan, Italy) (limit of detection on Coomassie stain SDS-PAGE of 250 ng) and FeSO_4_ (limit of detection for calcein 45.8 μg/dL) as standards).

### 2.4. Iron Quantitation

Iron content in samples and fractions was measured with the calcein methods. A mother stock of calcein (from Sigma-Aldrich, Milan, Italy) 1.6 mM was prepared in DMSO and stored at −20 °C protected from light. A working dilution of calcein 0.2 μM in 0.9% NaCl was freshly prepared before each measurement. Samples (Calibration curves, Elution fractions, immunoprecipitates) were precipitated and vacuum dried in a speedVac at R.T to induce release of bound iron. Then, 50 μL of 0.2 μM of calcein was added to each dried sample. Calcein fluorescence (λ_exc_ = 492 nm; λ_ems_ = 514 nm) was measured in a black optiplate (Perkin Elmer, Milan, Italy) in an Envision 2104 plate reader equipped with a monochromator, as already reported [19]. Titration curves and comparison with Atomic absorbance procedure (Supplementary Figure S1A) confirmed quenching of calcein fluorescence being less sensitive (limit of detection for calcein 45.8 μg/dL), however directly correlated to total iron concentration. 

### 2.5. Analysis of Protein Content

Protein content present in crude serum or in each of the eluted fractions was measured using the Bradford methods following manufacturer instructions. Briefly, 9 volumes of Bradford solution were added to 1 volume of sample and incubated for 15 min at R.T. Absorbance (λ = 525 nm) of the samples was measured using the spectrofluorometer Envision 2104 (Perkin Elmer). Gel filtration elution fractions were subjected to SDS-PAGE in accordance with the procedure of Laemmli (Laemmli, 1970), using a 12% resolving gel and 5% stacking gel in non-reducing condition (in the absence of reducing agent). The bands were visualized after staining with Coomassie Brilliant blue. 

### 2.6. Immuno-Isolation of Albumin from Serum Samples

For immuno-isolation of HSA and HSA-complexes, 50 μL of serum (storing of the sample at −80 °C and defrosting did not affect end results—Supplementary Figure S1B) was incubated with a polyclonal antibody against human albumin produced in goat (Invitrogen, Waltham, MA, USA, code AB_1954616, 1:10 dilution) for 2 h at 4 °C. Samples were then supplemented with 50 μL of a Protein A Sepharose suspension (10% d. *w*/*v* in 0.9% NaCl) and incubated on ice for a further 45 min. Protein A-antibody complexes were isolated by centrifugation at 30× *g* for 5 min in a tabletop centrifuge at 4 °C. Immuno-isolates were briefly washed in 0.9% NaCl and recentrifuged. Finally, immuno-isolates were resuspended in 0.9% NaCl and their iron and protein content were analyzed.

### 2.7. Statistical Analysis

Data are presented as mean value ± SEM. Statistical analysis was performed by one-way ANOVA followed by Bonferroni’s post hoc test for multiple comparisons, for both in vivo and in vitro experiments. The analysis was performed using GraphPad Prism 8 (GraphPad Software, San Diego, CA, USA) with a level of significance of *p* < 0.05.

## 3. Results

**Figure 1:**
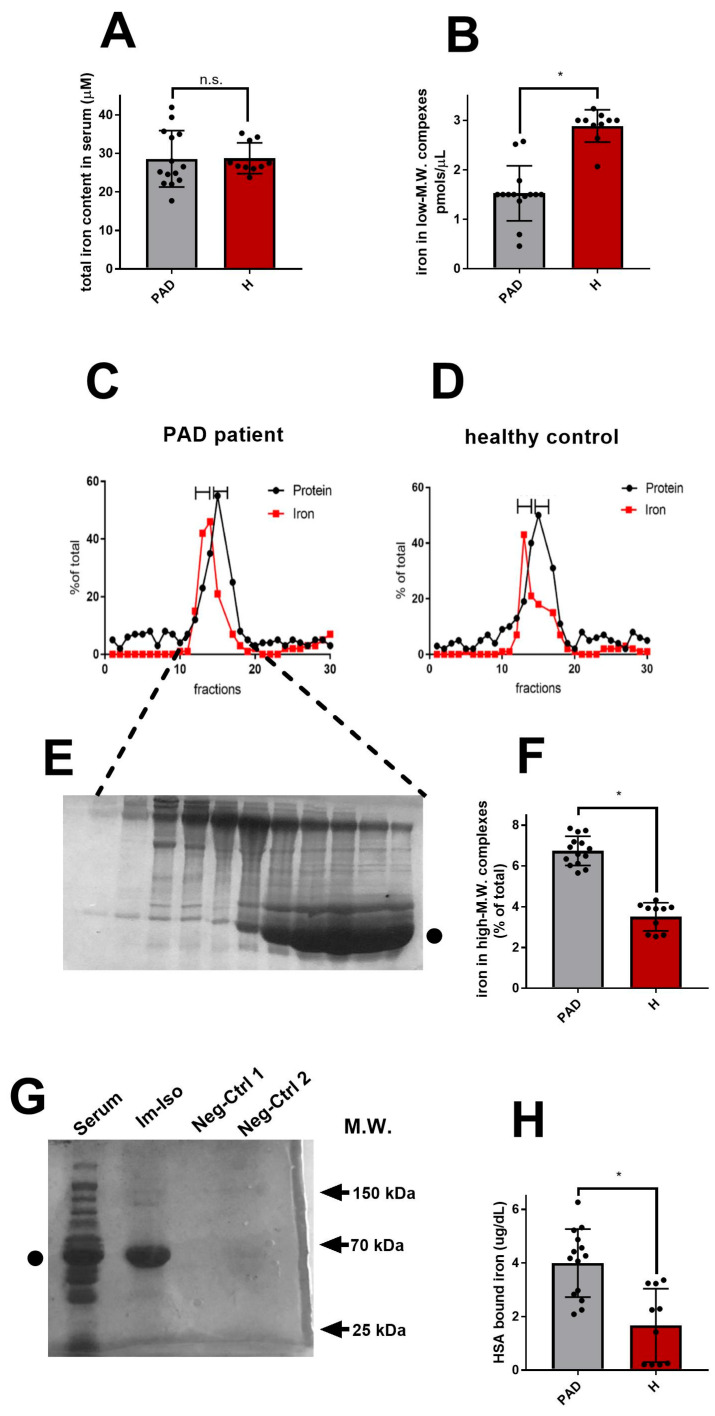
NTBI associates to HSA in PAD patients. (**A**) Total amount of iron measured in crude serum from PAD patients (PAD, gray bar) and healthy controls ((**H**), red bar). (**B**–**F**): Column-chromatographic procedures performed to isolate and analyze NTBI in PAD patients’ serum: (**B**) Iron measured in low M.W. complexes isolated by Desalting PD10-column in PAD patients (gray bar) and healthy controls (red bar); (**C**–**F**) Iron measured in high M.W. complexes isolated via ENRICH gel filtration column. Panels (**C**,**D**) show exemplificative results obtained for gel-filtration of serum from one of the PAD patients (**C**) and one healthy subject (**D**). For each elution fraction, iron content (red squares, calcein method) and protein content (black dots, Bradford methods) are indicated. In panel (**E**), Coomassie staining of non-reducing SDS-PAGE resolved elution fractions of sample shown in (**C**) (• indicates HSA). The bar graphs (**F**) show the average amount of iron measured in high-MW NTBI in PAD patients (gray bar) and in healthy controls (red bar). (**G**,**H**): Immunoisolation of iron-HSA complex. (**G**): Immunoisolates (Im-Iso) of one PAD patient were resolved on SDS-PAGE, Coomassie stained and compared to crude serum (serum), to a blank (immunoisolation in the absence of serum; neg ctrl 1) or to serum subjected to immunoisolation in the absence of anti-HSA antibody (only protein A; neg ctrl 2). The bar graph in (**H**) shows the average amount of iron measured in HSA immunoisolated complex of PAD patients (gray bar) vs. healthy controls (red bar). Data are presented as mean ± S.E.M. (n = 14, PAD patients; n = 10 healthy controls); * *p* value < 0.05 indicates statistical significance, n.s. non statistically different.

All the PAD patients (n = 14) enrolled in the study presented iron-related biochemical parameters falling in the normal physiological range (Table 1), including TIBC (Total Iron Binding Capacity), serum iron, ferritin, and transferrin values. As shown in Figure 1A, the total amount of iron in the serum of the above-mentioned patients was statistically similar to healthy controls (n = 10).

In order to detect any fluctuation in the pool of circulating NTBI in PAD patients, we analyzed their serum samples in a pipeline of different gel filtration techniques. Upon each chromatographic fractionation, iron and protein content in each fraction was measured by calcein fluorescence and Bradford assay, respectively. 

As first, we started isolating and measuring NTBI present in very low-molecular-weight (M.W.) complexes by using desalting columns. The latter allows rapid separation of high-M.W. complexes from low-M.W. substances. As shown in Figure 1B, the amount of NTBI present in low-M.W complexes was statistically lower in PAD patients compared to healthy controls, suggesting that PAD might reduce, rather than increase, the portion of NTBI chelated by small metabolites. 

We thus moved to isolate and analyze the pool of NTBI contained in high-M.W. complexes. Firstly, we analyzed proteins and protein complexes endowed with M.W. ranging from 30 to 650 kDa. As shown in Figure 1C–F, gel filtration indicated that, compared to healthy controls, PAD patients presented increased levels of iron associated with high-M.W. species. Proteins present in fractions containing the highest amount of iron were resolved (Figure 1E). Coomassie staining revealed the presence of HSA in these fractions. This result was confirmed by analyzing proteins and protein complexes ranging from 0.5 to 70 kDa (Supplementary Figure S1C,D). Compared to healthy control, PAD patients again presented increased levels of iron in fractions containing HSA. Despite the high amount of HSA co-eluted in the iron-containing fractions, we could not exclude by gel filtration that the iron was indeed binding to other proteins, including transferrin. Thus, in order to confirm the existence of an NTBI pool bound to HSA in PAD patients, we performed an immuno-isolation of HSA from the serum samples of PAD patients and healthy controls. Crude serum was incubated with a polyclonal antibody directed against human HSA. The HSA-antibody complexes were then isolated. Analyses on SDS-PAGE revealed that the immuno-isolation was successful, as shown by the purified band of HSA visible in the immuno-isolated sample (Figure 1G). Iron contents in immuno-isolates were measured (Figure 1H) and confirmed to be augmented in PAD patients compared to healthy controls.

## 4. Discussion

Peripheral arterial (PAD) and vascular (PVD) diseases are leading health concerns. PAD affects approximately 8.5 million Americans, and more than 200 million individuals worldwide, and its incidence is increasing due to obesity and diabetes [20]. Moreover, PAD is uncommon in patients under 50 years of age, but increases in prevalence with age and affects a substantial number of older adults [21]. A recent study evaluated the incidence and prevalence of PAD and critical limb ischemia in a large representative US sample of ≈12 million insured adults, based on insurance claims. From 2003 to 2008, the annual incidence of PAD and critical limb ischemia was 2.35% and 0.35%, respectively. Furthermore, the prevalence was 10.69% and 1.33% for PAD and critical limb ischemia, respectively [22]. Global data on trends in PAD prevalence between the years 2000 and 2010 have been recently published. Over that period, the number of individuals affected by PAD increased by 28.7% in low-income and middle-income countries and by 13.1% in high-income countries [20]. 

It is well known that NTBI exerts detrimental effects on cells and tissues [23]. By triggering Fenton and Haber-Weiss chemical reactions, iron not bound to transferrin generates oxygen radicals (ROS) [24]. ROS have clear and documented effects on many tissues and organs of the human body and their role in the etiology of CVD was postulated by Sullivan in 1981 [25] and is currently supported by much clinical evidence showing a higher incidence of cardiovascular disease in patients with iron overload [26,27,28]. Some authors have also postulated that oxidative stress could represent, more than the consequence, also a cause of the presence of NTBI-iron, with ROS promoting the release of NTBI-iron from iron-protein complexes, ferritin, and even from heme [9]. Nevertheless, the mechanism by which iron switches from transferrin to NTBI in PAD is still poorly investigated and hazy. Under physiological conditions the level of transferrin and its physiological saturation (20–35%) would be capable of sequestering large quantities of circulating NTBI. This is the reason why NTBI levels in normal healthy individuals do not generally exceed 1 μmol/L and the vast majority of studies on NTBI have been focused on patients presenting iron overload. In these patients, as a consequence of a higher transferrin saturation, a fraction of free iron escapes the specific carrier becoming NTBI. Surprisingly, high levels of NTBI have also been found in pathological conditions in the absence of iron overload: haemolytic anemia, acute coronary syndrome, liver disease, chronic renal failure, cancer, alcoholic liver disease [29,30,31,32,33]. The appearance of NTBI-iron in patients with normal transferrin saturation raises the question of how iron can remain free even in the presence of transferrin sites available for binding. In diabetes mellitus, it has been hypothesized that glycochelates, (glycates protein and peptides) might detach iron from transferrin and chelate it with a greater affinity [34,35]. 

Recently, fast and reliable protocols and methodologies to measure NTBI have been proposed [36]. In our study, in a small group of subjects affected by PAD, with and without diabetes mellitus, with normal values of serum iron, ferritin, and transferrin, we have shown that the pool of NTBI is mostly bound to HSA. HSA is a potent circulating antioxidant (the major extracellular source of reduced sulfhydryl groups) and a potent ROS-neutralizer [37]. Depending on its redox state, HSA can exist as mercaptalbumin, with a free thiol group on cysteine-34; nonmercaptalbumin-1 with cysteine-34 bound to homocysteine or glutathione; nonmercaptalbumin-2 with cysteine-34 oxidized to sulfinic or sulphonic acid [38,39]. HSA is able to eliminate multiple reactive species -of O_2_ and nitrogen, such as hydrogen peroxide (H_2_O_2_), peroxynitrite (ONOO^−^), superoxide (O_2_^•−^), and hypochlorous acid (HOCl) [40]. As already described, the NTBI-HSA complex is unstable and, as already reported in the literature [8], might act as an amplifier of oxidative stress and inflammation. Upon binding to metal ions (Cu^2+^/Fe^3+^), HSA promotes their reduction into more reactive and biologically more harmful states (Cu^+^/Fe^2+^) [41]. Reduction of iron by HSA seems to occur in the first minutes of coronary events. Some authors have shown that, rapidly before an ischemic event, an unusual form of HSA, called IMA (Ischemia Modified Albumin), is generated [42]. This modified form of HSA possesses an improved binding capacity for metal ions and, therefore, promotes the formation of dangerous free radicals [43]. A spectrophotometric test, the Albumin Cobalt Binding (ACB) test, has been developed to detect IMA and currently offers the possibility of improving the sensitivity of early diagnosis of myocardial ischemia (6–24 h earlier than troponin raise) [43]. Recently, the Food and Drug Administration (FDA) authorized the introduction, in laboratories, of the ACB test in routine analysis; there is in fact at least one automatic kit on the market for the ACB test by Ischemia Technologies (Denver, CO, USA). 

## 5. Conclusions

In conclusion, together with the identification of increased NTBI-HSA levels in the serum of PAD patients, we have developed here a rapid method to isolate NTBI-HSA from other forms of serum iron. Despite the NTBI-has, an immunoisolation procedure has to be performed on a larger cohort of patients; its future validation might open new frontiers in the prevention and treatment of PADs. All the volunteers of our study were Caucasian. It will be interesting to measure the amount of NTBI-HSA complex in volunteers of other ethnicities. People of African origin, for example, are affected by iron overload disease more than Caucasians. Moreover, as suggested by Patel et al. [9], it will be interesting to measure NTBI-HSA complexes in patients affected by diseases other than iron-overload- disease, to verify their contribution to other pathological conditions. HSA is present at high concentrations in the cerebrospinal fluid, where it plays an important role in redox balance [44]. While our small study focused on vascular patients, the hypothetical role of NTBI in brain and neurodegenerative pathologies as well as a possible interconnection between peripheral vascular pathologies and neurodegeneration, certainly represents a future goal of investigation.

The test we have developed could be used as well to measure NTBI-HSA complex in healthy patients, to verify the influence of their living habits (diet, smoking, physical exercise) on the NTBI pool. Smoking, for example, does promote oxidative stress by increasing circulating ROS and weakening the antioxidant defense system [45]. Indeed, while many studies have highlighted a greater level of lipid peroxidation in smokers vs. non-smokers, none of these studies were aimed at detecting altered levels of NTBI in one of the two groups.

## Figures and Tables

**Table 1:** Iron-related biochemical parameters of patients enrolled in the study.

Demographics	PAD (n = 14)	H (n = 10)	Normal Value Range
Age (years)	73.2 (47–92)	60.1 (25–69)	
Male sex (No. (%))	10 (71%)	8 (80%)	
White ethnicity (No. (%))	14 (100%)	10 (100%)	
**Clinical Parameters**			
Diabetes	7 (50%) *	0 (0%)	
Hypertension	13 (93%) *	0 (0%)	
Dyslipidemia	11 (79%) *	3 (30%)	
Cigarette Smoking	1 (7%) *	5 (50%)	
Obstructive arteriopathy (No. (%))	11 (79%) *	0 (0%)	
CVI (No. (%))	3 (21%) *	0 (0%)	
Serum Iron (µg/dL)	154 (115–176)	121 (91–158)	(60–170)
Serum Transferrin (g/L)	2.9 (2.6–3.2)	2.8 (2.6–3.1)	(2.5–3.8)
Serum Ferritin (ng/mL)	75.2 (22–134)	91.3 (38–156)	(22–322)
Total Iron Binding Capacity (µg/dL)	296.2 (273–350)	302.4 (260–380)	(240–450)

Values are expressed as means and value range. Abbreviations: PAD, peripheral arterial disease; H, healthy controls; CVI, chronic venous insufficiency. (* = *p* value < 0.05 vs. H).

## Data Availability

The data used to support the findings of this study are included in the article.

## Supplementary Materials

The following supporting information can be downloaded at: https://www.mdpi.com/article/10.3390/antiox12020503/s1, Figure S1: Supplementary Methodological Information.
