# Supplementary material for: Increased Levels of Circulating Iron-Albumin Complexes in Peripheral Arterial Disease Patients
Source: Antioxidants (Basel). 2023 Feb 16;12(2):503. doi: 10.3390/antiox12020503 (PMC9952351; doi:10.3390/antiox12020503)
Supplement: Supplementary file 1 [file antioxidants-12-00503-s001.zip › antioxidants-2153803-supplementary.pdf]

Supplementary Information

Increased levels of circulating Iron-Albumin complexes in peripheral arterial disease patients

Elisabetta Schiano [1], Enrico Cappello [2], Domenico Cecere [2], Francesco Pompeo [2], Ettore Novellino [3], Mariano Stornaiuolo [1*] & Marcello Izzo [4*].

[1] Department of Pharmacy, University of Naples Federico II, Via Montesano 49, 80131 Naples, Italy
[2] IRCCS Neuromed, 86077 Pozzilli, Italy
[3] Department of Medicine and Surgery, Università Cattolica del Sacro Cuore, 00168 Rome, Italy
[4] MathTechMed-Department of Mathematics for Technology, Medicine and Biosciences Research Center, University of Ferrara, 44121 Ferrara, Italy

* Correspondence: mariano.stornaiuolo@unina.it (M.S.); zzimcl@unife.it (M.I.)

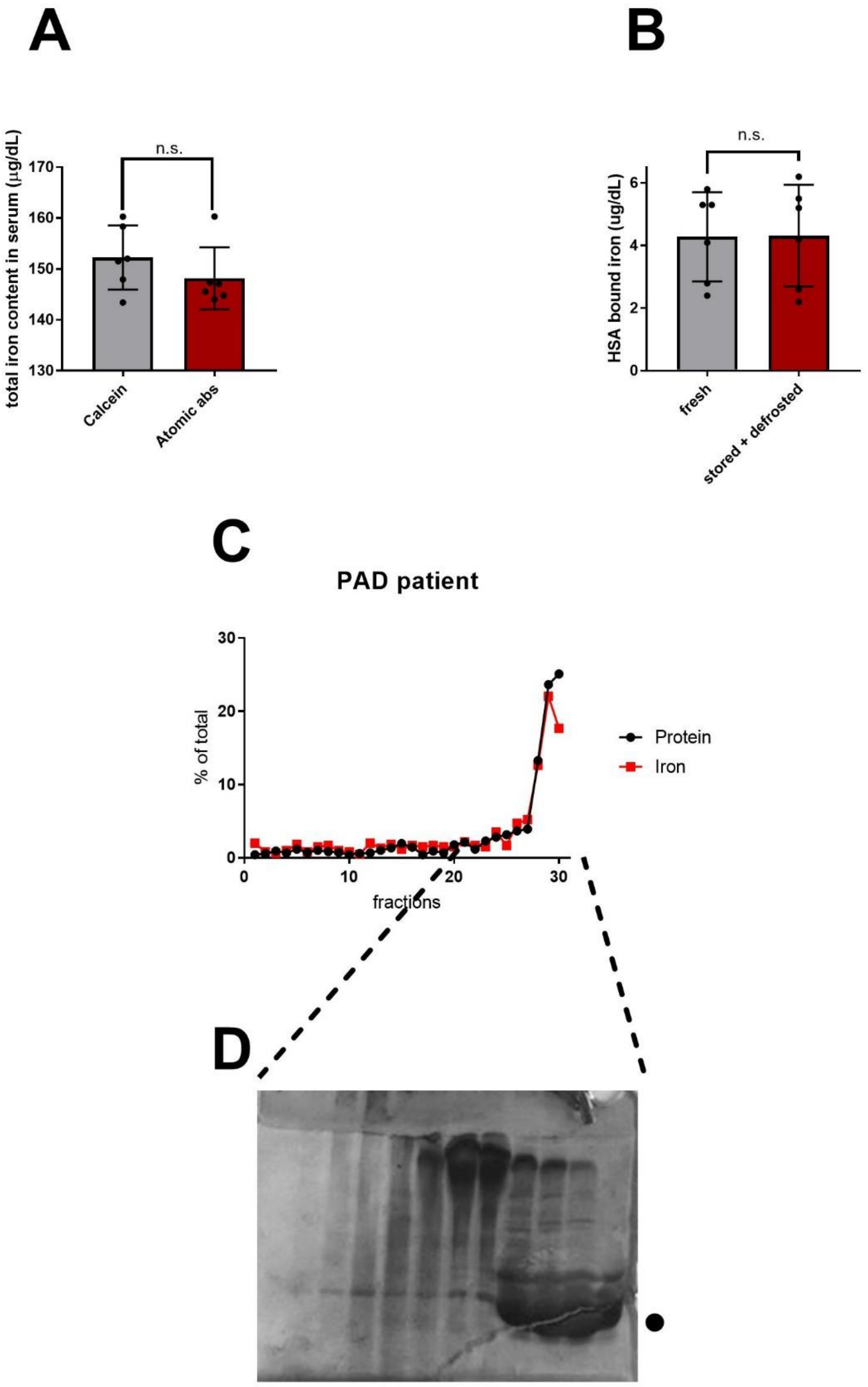

Figure S1. **Supplementary Methodological Information. (A)** Comparison between iron measurement by calcein (gray bar) and atomic absorbance spectroscopy (red bar) (n=6, n.s. non statistically different). Despite less sensitive (limit of detection of 45.8 µg/dL and 2 ppb for calcein and atomic absorbance spectroscopy, respectively) calcein quenching directly correlates to total iron concentration. **(B)**: Effect of freezing and defrosting on immunoisolation of NTBI-HSA complex. Amount of iron (calcein method) present in HSA immunoisolated from serum samples subjected (red bar) or not (gray bar) to freezing and defrosting (n=6, n.s. non statistically different). **(C-D)**: Panels C and D show exemplificative results obtained for gel-filtration of serum from one of the PAD patients on Enrich SEC 70-75 HR 10/300 column. For each elution fraction, iron content (red squares, calcein method) and protein content (black dots, Bradford methods) are indicated. In panel D, Coomassie staining of non-reducing SDS-PAGE resolved elution fractions of sample shown in C (• indicates HSA).
